# Improved sensitivity for detection of breast cancer by combination of miR-34a and tumor markers CA 15-3 or CEA

**DOI:** 10.18632/oncotarget.25077

**Published:** 2018-04-27

**Authors:** Martin Zaleski, Makbule Kobilay, Lars Schroeder, Manuel Debald, Alexander Semaan, Karina Hettwer, Steffen Uhlig, Walther Kuhn, Gunther Hartmann, Stefan Holdenrieder

**Affiliations:** ^1^ Institute of Clinical Chemistry and Clinical Pharmacology, University Hospital Bonn, Bonn, Germany; ^2^ Department of Gynecology and Obstetrics, University Hospital Bonn, Bonn, Germany; ^3^ Center for Integrated Oncology (CIO) Köln/Bonn, Bonn, Germany; ^4^ Department of Surgery, University Hospital Bonn, Bonn, Germany; ^5^ QuoData Statistics, Dresden, Germany; ^6^ Joint Research and Services Center for Biomarker Evaluation in Oncology, Bonn/Dresden, Germany

**Keywords:** breast cancer, miRNA, miR-34a, CEA, CA 15-3

## Abstract

**Background:**

MicroRNAs biomarkers have shown value for diagnosis and prognosis of various cancers. Combination with established tumor markers has rarely been done.

**Results:**

Breast cancer patients had significantly higher serum RNA loads (AUC 0.665), lower miR-34a (AUC 0.772), higher CEA and CA 15-3 levels (AUCs 0.717 and 0.721) than healthy controls. miR-34a correlated with tumor stage and hormone receptor status. There was no significant difference between groups for all other miRNAs. Combination of miR-34a with CEA or CA 15-3 led to improved AUCs of 0.844 and 0.800, respectively. Sensitivity of miR-34a and CA 15-3 reached 56.1% at 95% specificity. When compared with benign breast diseases, combination of miR-34a (AUC 0.719) and CEA (0.623) or CA 15-3 (0.619) resulted in improved performances (0.794 and 0.741). Sensitivity of miR-34a and CA 15-3 reached 53.7% at 95% specificity.

**Conclusion:**

While miR-34a provides valuable information for diagnosis and staging, combination with tumor markers CA15-3 or CEA improves the sensitivity for breast cancer detection.

**Patients and Methods:**

The diagnostic relevance of the miR-21, miR-34a, miR-92a, miR-155, miR-222 and miR-let-7c was tested in sera of 103 individuals (55 breast cancer, 20 benign breast diseases, 28 healthy controls). MiRNAs were detected by quantitative rt-PCR after extraction and reverse transcription. Cel-miR-39 and miR-16 were used for normalization. Established tumor markers CEA, CA 15-3, CA 19-9 and CA 125 were measured by automatized immunoassays. Diagnostic performance was tested by areas under the curve (AUC) of receiver operating characteristic (ROC) curves and sensitivities at 90% and 95% specificity.

## INTRODUCTION

Breast cancer is by far the most frequently diagnosed cancer and cause of cancer death among women. There were an estimated 1.7 million new cases (25% of all cancers in women) and 0.5 million cancer death (15% of all cancer deaths in women) in 2012 [1]. Improved diagnostic tools that enable an earlier and more sensitive detection have led to a better outcome for patients and also to economic advantages [2]. However, screening methods like mammography and breast examination still miss 10–40% of early breast cancer. Especially for young women, whose tumors are often more aggressive, the existing cancer screening tools are less effective due to their dense breast tissue structure [3, 4]. Despite the benefits of mammography, exposure to ionizing radiation, costs and follow-up exams of false positive findings have to be considered as limiting factors [5]. Therefore, highly sensitive and specific blood-based biomarkers would be highly useful to improve breast cancer detection.

Established tumor markers such as carcinoembryonic antigen (CEA) and carbohydrate antigen 15-3 (CA15-3) are only valuable in late stages and support therapy response assessment and early detection of recurrent disease. In early stages, their sensitivity is limited [6–9]. During the last years, microRNAs have come up as a promising new biomarker class. They are single-stranded, non-coding RNA species with around 18–24 nucleotides, regulate the expression of diverse oncogenic genes and play a major role for many cellular functions, like cell cycle, development, differentiation, proliferation and apoptosis. Thereby, each miRNA targets multiple different mRNAs and each mRNA is regulated by diverse miRNAs producing a very complex web of tight regulation. Dysregulated expression of specific miRNAs affects many onco- and suppressor genes that influence cancer initiation, progression and metastasis [10]. Up to now, more than 2000 miRNAs have been identified in human tissue. Beyond cancer diseases, they are described to be also relevant in traumatic, ischemic, inflammatory and degenerative diseases [11]. Therefore, cancer specificity has to be shown not only on the basis of healthy controls but also on all pathologies that have to be considered in differential diagnosis.

MiRNA deregulation in breast cancer was first demonstrated by Iorio *et al*. in 2005 [12]. Other research projects showed that cancer-related miRNAs are present in blood serum [13–15]. Further it was reported that miRNAs are circulating in a cell-free form in blood of healthy and cancer bearing individuals and that miRNAs are protected from degradation by RNAses [16–18]. While it was assumed that circulating RNAs (including miRNAs) are rapidly destroyed accumulated data showed that miRNAs in serum are quite stable when exposed to external influences like pH-alteration, storage, freezing and thawing [19–21]. Reasons for their stability may be the association with exosomes or the binding to argonaut proteins or high-density lipoproteins [21].

In breast cancer patients, some miRNAs such as miR-21 or miR-155 were found to be elevated in serum [22–26], others were decreased like miR-34a [23, 27]. In addition, correlation with tumor stage was described for miR-21 and miR-34a [23–25]. However, there were also contradictory reports about the same miRNAs challenging the previous findings [28–31]. Therefore, we here addressed the thorough investigation of a panel of circulating miRNAs (miR-16, miR-21, miR-34a, miR-92a, miR-155, miR-222 and let-7c) as potential serum biomarkers for the detection of breast cancer and compared them with healthy women and patients with benign breast cancer as control groups. Importantly, the results were compared with the established tumor markers CEA and CA 15-3 as well as with CA 19-9 and CA 125 in order to see a potential additive value of the miRNAs.

## RESULTS

**Figure 1:**
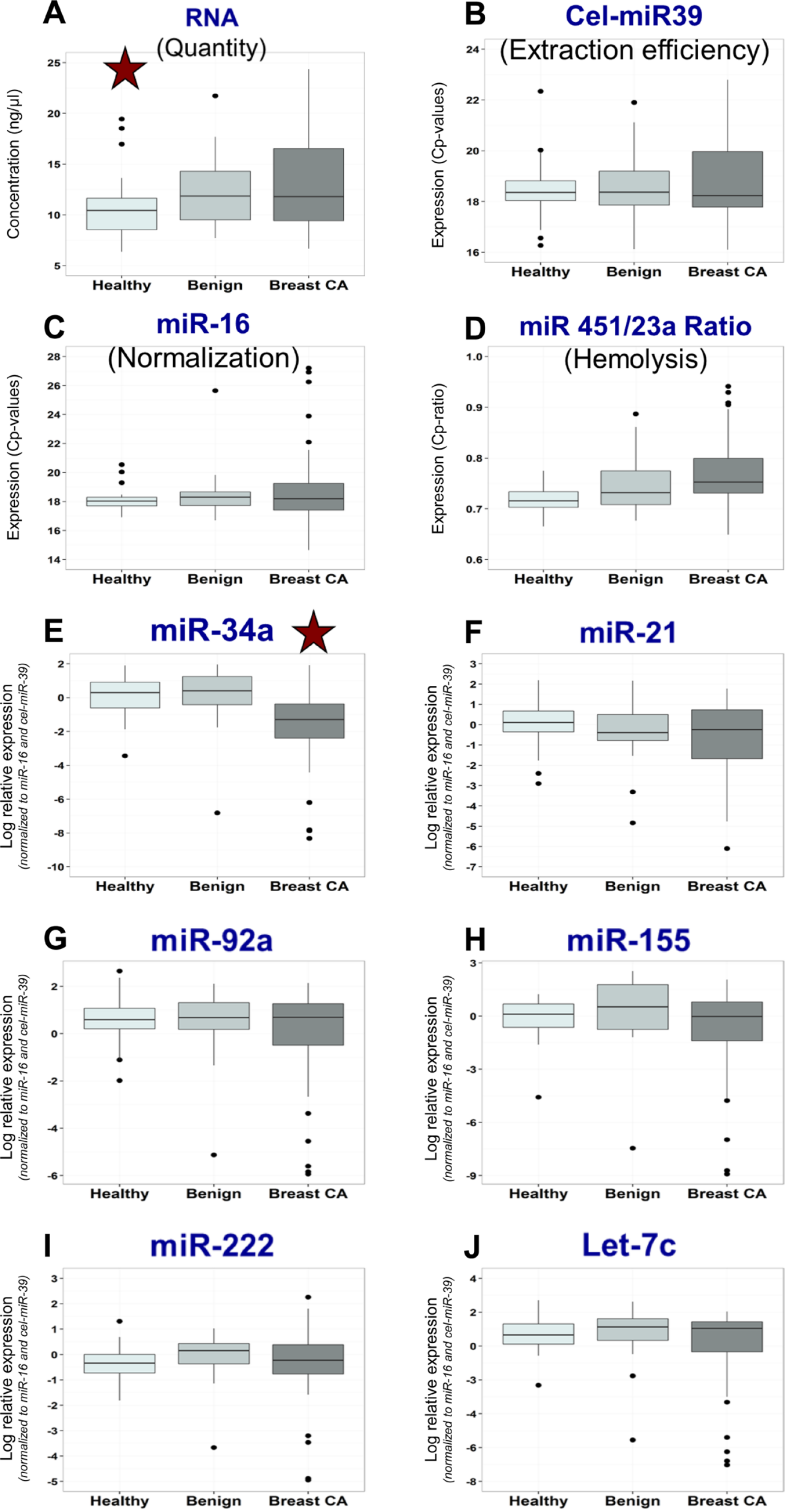
Distribution of miRNA biomarker levels in various patient groups Box plots for RNA quantity (**A**) and for miRNA biomarkers cel-miR-39 (**B**; extraction efficiency), miR-16 (**C**; normalization), miR 451/23a ratio (**D**; hemolysis indicator), miR-34a (**E**), miR-21 (**F**), miR-92a (**G**), miR-155 (**H**), miR-222 (**I**) and miR-let-7c (**J**) indicating medians, means, interquartile ranges, whiskers and outliers for the groups of healthy individuals, patients with benign and malignant breast diseases.

Methodical performance was good for all miRNA rtPCR assays with PCR efficacies close to 2.0, excellent calibration curves as well as low intra- and interassay imprecisions. RNA quantity after extraction was quite different in the diagnostic groups with higher levels in patients with breast cancer and benign breast diseases as compared with healthy controls. Extraction and transcription efficiency was comparable in all groups. However, there were considerable variations within each group. Similarly, miR-16 that was used for normalization of miR-values showed comparable median values and distributions in all groups with considerable intra-group variations. Ratio of miR-451 to miR-23a that was reported to indicate hemolytic contamination showed a tendency to higher values in cancer patients; however, this was not significant. As most of the Ct-ratio values were below 5 it can be assumed that hemolysis was not a relevant factor that could have influenced the clinical results. Among all other microRNAs investigated, miR-34a serum levels were significantly lower in breast cancer patients as compared with healthy controls and also than patients with benign breast diseases. For the other microRNAs miR-21, miR-92a, miR-155, miR-222 and miR-let-7c, no differences in relative levels between the differential diagnostic groups were observed (Figure 1).

**Figure 2:**
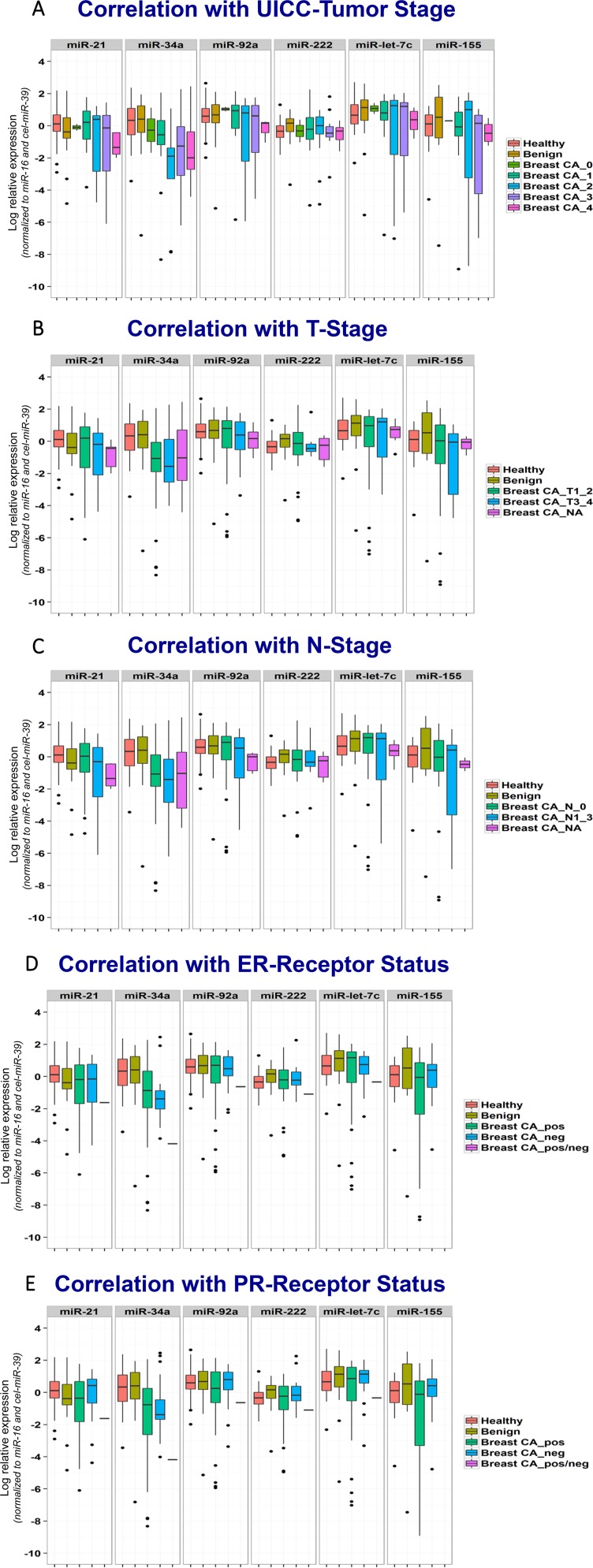
Correlation of miRNA biomarkers with clinical features of breast cancer patients Box plots for miRNA biomarkers miR-21, miR-34a, miR-92a, miR-222, miR-let-7c and miR-155 indicating medians, means, interquartile ranges, whiskers and outliers for the groups of healthy individuals, patients with benign and malignant breast diseases differentiated according to UICC tumor stage (**A**), T-stage (**B**), N-stage (**C**), estrogen receptor (ER)-status (**D**), and progesterone receptor (PR)-status (**E**). MiRNA are normalized to miR-16 and cel-miR-39.

In subgroup analyses, miR-34a levels correlated with UICC-tumor stage and hormone receptor status. Regarding UICC-stage, patients with stage II and higher stage tumor disease had significantly lower levels. However, there was no difference with regard to tumor size or lymph node status: Similar values were obtained for T1-2 and T3-4 stage patients as well as for N0 and N1-3 stage patients. Patients with negative ER or PR receptor status had somewhat lower values than receptor positive patients. For all other miRNA markers, no differences according to tumor stage or receptor status were seen (Figure 2).

**Figure 3:**
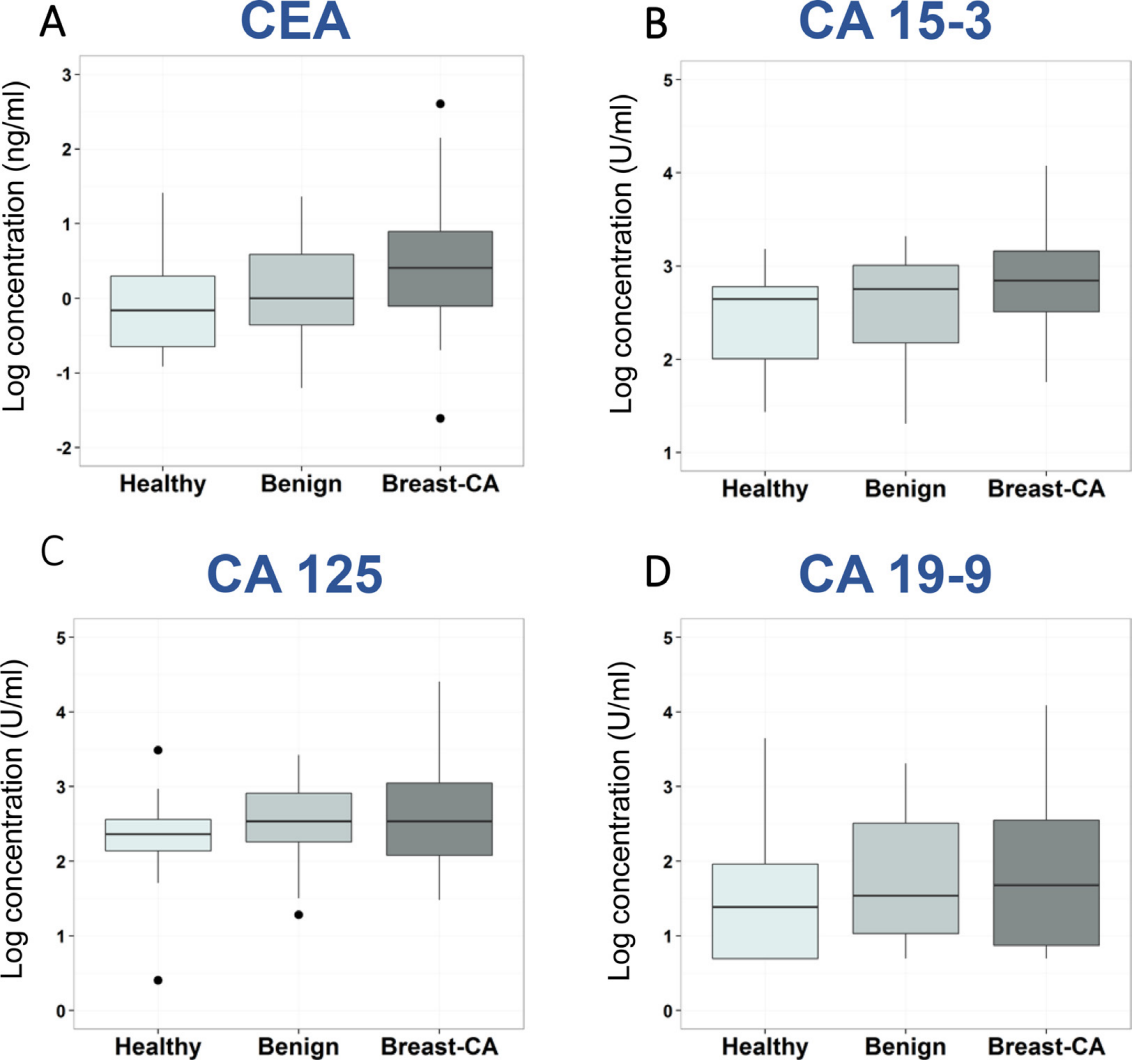
Distribution of tumor marker levels in various patient groups Box plots for CEA (**A**), CA 15-3 (**B**), CA 125 (**C**), and CA 19-9 (**D**) indicating medians, means, interquartile ranges, whiskers and outliers for the groups of healthy individuals, patients with benign and malignant breast diseases.

As expected, tumor markers CEA and CA 15-3 were significantly higher in serum of breast cancer patients as compared with healthy women, however, not as compared to patients with benign breast diseases. For CA 125 and CA 19-9, no differences were found in the different patient groups (Figure 3).

**Figure 4:**
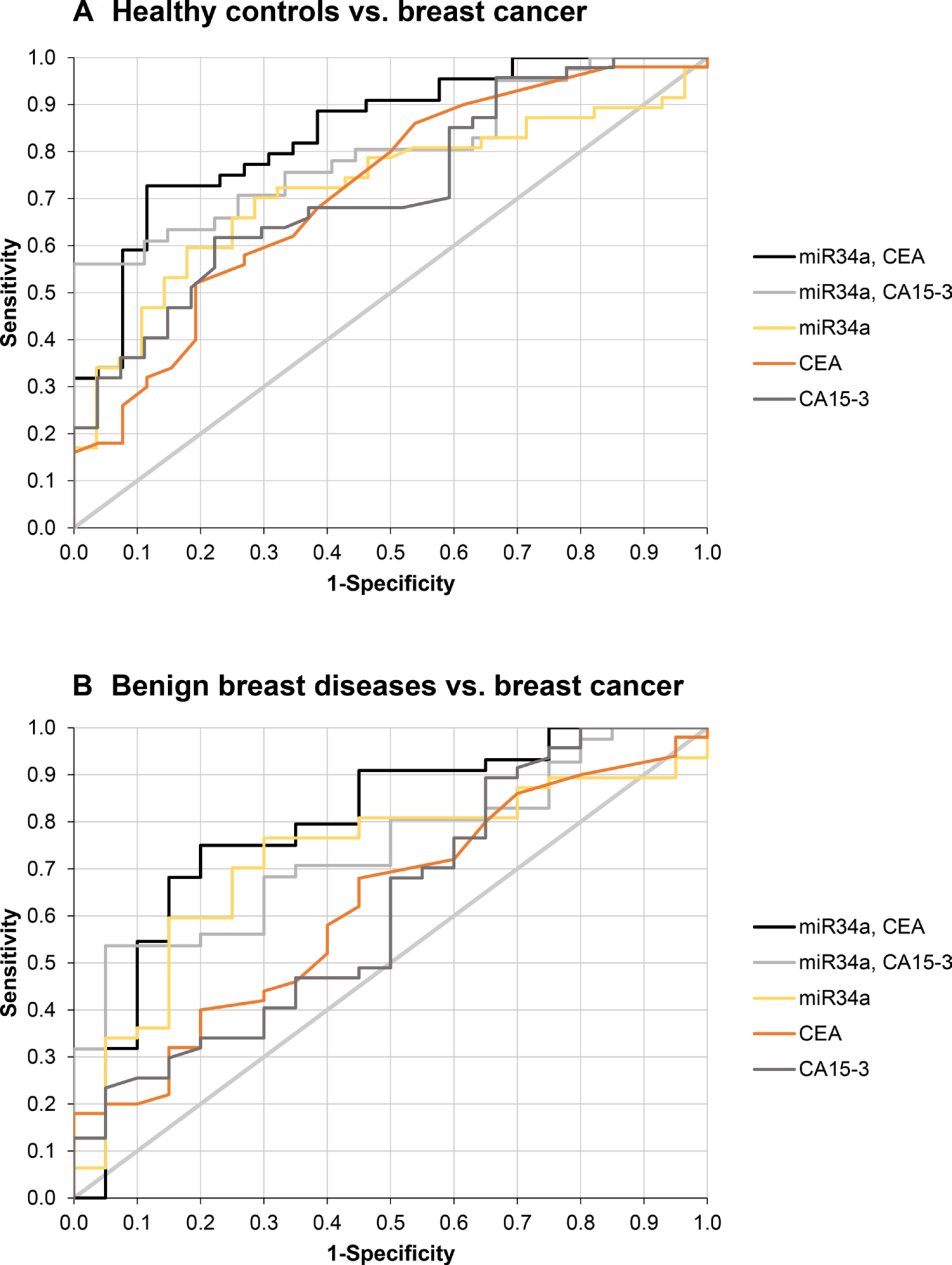
Discriminative power of significant biomarkers between breast cancer and control groups Receiver operating characteristic (ROC) curves provide a sensitivity-specificity profile over the whole range of possible cutoffs for the discrimination between breast cancer and healthy controls (**A**) as well as between breast cancer and benign breast diseases (**B**).

Concerning the differentiation between breast cancer patients and healthy women, the markers RNA quantity (AUC 0.665; 95% CI 0.546–0.783), miR-34a (AUC 0.722; 0.608–0.836), CEA (AUC 0.717; 0.601–0.833) and CA 15-3 (AUC 0.721; 0.605–0.837) showed good diagnostic performance. Sensitivities at 95% specificity were 14.5% for RNA, 34.0% for miR-34a, 18.0% for CEA and 31.9% for CA 15-3. While combination of CEA and CA 15-3 led only to a slightly higher AUC of 0.741 (0.628–0.854) and slightly higher sensitivities of 38.5% at 90% and 95% specificities, the combination of miR-34a with CEA or CA 15-3 improved the performance considerably with AUCs of 0.844 (0.754–0.933) and 0.800 (0.697–0.904), respectively. Sensitivities of the combinations reached for miR-34a and CEA 59.1% and 34.1% at 90% and 95% specificities for breast cancer detection, and for miR-34a and CA 15-3 56.1% for both 90% and 95% specificities (Figure 4; Table 1).

**Table 1:** Performance of biomarkers for discrimination of patient groups

Marker	*N* CA	*N* Controls	AUC	95% CI lower	95% CI upper	Sens at 95% Spec	Sens at 90% Spec
**Comparison: Healthy controls vs. breast cancer**							
RNA	55	28	0.665	0.546	0.783	14.5%	21.8%
miR 21	55	28	0.583	0.455	0.711	16.4%	21.8%
**mir 34a**	**47**	**28**	**0.722**	**0.608**	**0.836**	**34.0%**	**36.2%**
mir 92	55	28	0.464	0.331	0.597		
mir 222	55	28	0.534	0.403	0.665	12.7%	16.4%
mir let 7c	55	28	0.512	0.380	0.644		12.7%
mir 155	33	19	0.525	0.361	0.689	16.7%	24.2%
**CEA**	**50**	**26**	**0.717**	**0.601**	**0.833**	**18.0%**	**26.0%**
**CA 15-3**	**47**	**27**	**0.721**	**0.605**	**0.837**	**31.9%**	**36.2%**
CA 19-9	50	27	0.616	0.488	0.744	14.0%	24.0%
CA 125	47	27	0.604	0.473	0.735	29.8%	34.0%
(CEA) + (CA 15-3)	47	26	0.741	0.628	0.854	38.5%	38.5%
**(miR 34a) + (CEA)**	**44**	**26**	**0.844**	**0.754**	**0.933**	**34.1%**	**59.1%**
**(miR 34a) + (CA15-3)**	**45**	**23**	**0.800**	**0.697**	**0.904**	**56.1%**	**56.1%**
**Comparison: Benign breast diseases vs. breast cancer**							
RNA	55	20	0.515	0.367	0.663	20.0%	23.6%
miR 21	55	20	0.486	0.337	0.635		
**mir 34a**	**47**	**20**	**0.719**	**0.594**	**0.844**	**34.0%**	**36.2%**
mir 92	55	20	0.425	0.275	0.575		
mir 222	55	20	0.586	0.444	0.728	16.4%	20.0%
mir let 7c	55	20	0.563	0.419	0.707	14.5%	23.6%
mir 155	33	10	0.603	0.409	0.797	6.1%	30.3%
**CEA**	**50**	**20**	**0.623**	**0.483**	**0.763**	**20.0%**	**20.0%**
**CA 15-3**	**47**	**20**	**0.619**	**0.477**	**0.761**	**23.4%**	**25.5%**
CA 19-9	50	20	0.514	0.364	0.664	6.0%	14.0%
CA 125	47	18	0.521	0.364	0.678	19.1%	19.1%
**(miR 34a) + (CEA)**	**44**	**20**	**0.794**	**0.685**	**0.904**	**31.8%**	**54.5%**
**(miR 34a) + (CA15-3)**	**45**	**20**	**0.741**	**0.620**	**0.863**	**53.7%**	**53.7%**

Performance of biomarkers and combinations thereof is given by the area under the curve (AUC) or receiver operating characteristic (ROC) curves with corresponding 95% confidence interval limits as well as sensitivities for cancer detection at specificities of 95% and 90%.

In the comparison of breast cancer and benign breast diseases that is more relevant for differential diagnosis, the diagnostic performance of miR-34a (AUC 0.719; 0.594–0.844) and the sensitivity of 34.0% at 95% specificity remained at a high level. In contrast performances of CEA (AUC 0.623; 0.483–0.763) and CA 15-3 (AUC 0.619; 0.477–0.761) dropped down. Most impressively, the combination of miR-34a with CEA or CA 15-3 resulted in a considerably improved performance with AUCs of 0.794 (0.685–0.904) and 0.741 (0.620–0.863), respectively. Sensitivities were 54.5% and 31.8% at 90% and 95% specificities for the combination of miR-34a and CEA, and 53.7% for both specificities for the combination of miR-34a and CA 15-3, respectively (Figure 4; Table 1).

## DISCUSSION

MicroRNAs have shown to be promising biomarkers for the detection of cancer diseases. Different compartments such as serum, plasma, exosomes or tumor cells themselves were discussed as sources for informative changes of tumor cell metabolism or altered reaction of immune and stroma cells upon tumor growth and invasiveness [10, 11]. As microRNAs were reported to be stable under varying preanalytical conditions they are considered as potential future tumor markers [19, 20, 32]. While several studies have shown the differential diagnostic power of miRNAs for different tumor diseases, only some have used benign diseases of the respective organ as control group and not only healthy individuals [33]. Some markers have found to be involved in the regulation of tumor growth and metastasis pathways such as miR-21 or miR-34 [24–25, 34–53]. However, there were discrepant results when these markers were used as diagnostic tools – even in the same tumor entities [22, 28–31]. Moreover, only few studies have done a thorough comparison with the already established tumor markers to investigate whether miRNAs have superior performance or can at least add to the diagnostic sensitivity [54–56]. Here we assessed the differential diagnostic power of a panel serum miRNAs (miR-16, miR-21, miR-34a, miR-92a, miR-155, miR-222 and let-7c) for the detection of breast cancer that have been described earlier to be relevant for this purpose [22, 23, 26, 28, 34–53, 57–72]. We compared their expression in sera of breast cancer patients in relation to healthy women and patients with benign breast diseases as control groups. Finally, results were correlated with the established breast tumor markers CEA and CA 15-3 as well as with CA 19-9 and CA 125 in order to see a potential additive value of the miRNAs.

Among all microRNAs investigated, only miR-34a serum levels were able to discriminate significantly between breast cancer patients and healthy controls and also when compared with patients with benign breast diseases. In addition, miR-34a levels correlated with UICC-tumor stage and hormone receptor status. For all other microRNAs miR-21, miR-92a, miR-155, miR-222 and miR-let-7c, no differences in relative levels between the differential diagnostic groups were found.

The finding of lower miR-34a levels in breast cancer, particularly in advanced stages, is in line with other studies that reported downregulated miR-34a expression in sera of breast cancer patients and lower miR-34a levels in higher UICC stage patients, too [23]. In addition, it was shown that low expression of miR-34a in FFPE-tissue samples was strongly associated with poor outcome of breast cancer patients [27]. However, there were also discrepant findings demonstrating higher miR-34a expression in breast cancer patients as compared with healthy controls, in advanced stage tumors and in hormone-receptor negative cancers [22, 28]. These differences may be explained by the size and selection of patient groups in the studies and by different methodical procedures that may influence the results considerably. From a biological point of view, our results are in line with the tumor suppressive function of miR-34a. During tumorigenesis and progression miR-34a is downregulated abrogating its function to induce cell cycle arrest and p53-dependent apoptosis of tumor cells [39, 40]. MiR-34a exerts its regulative role for cell cycle, differentiation, apoptosis, cancer cell progression and metastasis by targeting genes such as SIRT1, Bcl-2 and HMGI-C [41–47, 73, 74]. In addition, it is involved in mediating immune paralysis of T-lymphocytes via the PD1/PD-L1 system [48–52]. Similar to breast cancer, miR-34a was found to be downregulated also in other tumor entities such as non-small cell lung cancer, colon cancer, pancreatic cancer, neuroblastoma and leukemia [75–82]. The diagnostic role of microRNA-34a in breast cancer was also assessed by a recent systematic review and meta-analysis [83].

Unfortunately, the great expectations set in miR-21, that is known to stimulate cell invasion and metastasis in breast and other cancers, as a diagnostic serum biomarker of breast cancer were not met in our study. Several studies reported higher miR-21 expressions in the sera of breast cancer patients than in healthy controls as well as a correlation with advanced tumor stage, lymph node metastasis and poor prognosis [24, 25, 37, 38]. However, others could not confirm these positive results [29–31]. Similarly discrepant results are described for miR-155 in the literature. MiR-155 is known to be a potent suppressor of apoptosis and to target the tumor suppressor gene “suppressor of cytokine signaling 1(SOCS1)” [57, 58]. While some studies reported elevated levels of miR-155 in serum of breast cancer patients [22, 23, 26], others could not confirm these findings [28–30]. Further miRNAs such as miR-92a and miR-222 that are involved in cancer pathogenesis [59, 64–66] and that were considered useful for the detection of breast, but also gastrointestinal cancers have controversely been discussed in the literature so far [24, 63]. Indeed, miR-222 is supposed to increase cell migration in the epithelial to mesenchymal transition acting downstream of the RAS-RAF-MEK oncogenic pathway [65, 67] and to be a promising therapeutic target for estrogen-receptor downregulated breast cancer [68]. However, we could not confirm earlier findings of elevated miR-222 serum levels in breast cancer patients [69]. Tumor suppressing miR-let-7c that downregulates the oncogene RAS function in cellular physiological conditions and breast cancer cells [70, 71] was no potential diagnostic marker in our setting, too.

As differences in the outcome of diverse studies may be explained by variations in the preanalytical and analytical part of microRNA assessment we performed a thorough schedule for sample collection and handling, establishment of the methods as well as various quality controls through all analytical steps: Samples from patients and controls were collected according to SOPs of the Biofluid Biobank of the University Hospital Bonn and were stored under controlled conditions at -80°C until marker measurement. Assays for miRNA determination were set up according to MIQE criteria and quality controls for RNA extraction, transcription (spiked in exogenous synthetic control miRNA cel-miR 39), quantification (diverse controls, duplicates) and interpretation (normalization by miR-16) were used [28, 34]. Samples of all diagnostic groups were mixed in all runs to minimize interassay variability. Potential influence of contamination by blood cells and hemolysis in serum samples was controlled by the ratio of miR-23a and miR-451 as suggested by Blondal *et al*. [32] Finally, results were calculated by independent professional biostatisticians who took quality aspects of miRNA determination and value distribution into consideration to avoid bias and overfitting to the present sample. Of course, the evaluation has exploratory nature as the number of the patients investigated was limited. However, it gives a robust basis for further validation studies.

Importantly, the power of established tumor markers was assessed in the same patient setting as well. Thereby, the breast tumor markers CEA and CA 15-3 showed good performances for differential diagnosis of breast cancer from healthy women and slightly lower sensitivities when patients with benign breast diseases were used as control groups. For CA 125 and CA 19-9, no differences were found in the different patient groups. AUC of ROC curves for breast cancer detection vs. healthy women yielded similar results for miR-34a, CEA and CA 15-3 and sensitivities at 95% specificity were best for miR-34a and CA 15-3. When patients with benign breast diseases were considered as control groups, miR-34a was clearly superior to CEA and CA 15-3 alone. These results are in line with other studies that reported similar results for CA 15-3 for breast cancer detection [84].

Most impressively, we found considerably improved performances for discrimination of breast cancer patients from either control group when miR-34a was combined with one of the tumor markers CEA or CA 15-3 resulting in higher AUCs and sensitivities at high specificities that are requested for differential diagnosis. This shows that miR-34a and cancer cell surface markers such as CEA and CA 15-3 play different roles in breast cancer development and provide additive diagnostic information. To our knowledge this is the first evidence that a combined approach of miRNAs and established tumor markers leads to an improved diagnostic sensitivity in breast cancer detection that warrants consideration by further validation studies.

## PATIENTS AND METHODS

### Patients

In the present study, serum samples from 103 caucasian women were analyzed. Among them were 55 patients with breast cancer (2 *in situ* cancer, 20 Union for International Cancer Control (UICC) stage I, 12 stage II, 14 stage III, and 7 stage IV), 20 patients with benign breast diseases (ductal hyperplasia, mastopathy, mastitis, etc.) and 28 healthy women as control group. Samples were taken from all patients at time of active disease and before undergoing any therapeutic procedure at the University Hospital Bonn. The samples of the healthy control group were obtained from women, who had no history of cancer and were in good health on the basis of self-report. Detailed patient characteristics are listed in Table 2.

**Table 2:** Patient characteristics

Groups	*N*	Age (median/range)
Healthy controls	28	44 (20.1–64.5)
Benign breast diseases	20	54 (24.2–81.8)
Breast cancer	55	59 (32.6–85.8)
**UICC stage**	**N**	**Percentage**
0	2	3,6%
1	20	36,4%
2	12	21,8%
3	14	25,5%
4	7	12,7%
**T-stage**	**N**	
is	2	3,6%
1	24	43,6%
2	17	30,9%
3	5	9,1%
4	7	12,7%
**N-stage**	**N**	
0	42	76,4%
1	6	10,9%
2	5	9,1%
3	2	3,6%
**M-stage**	**N**	
0	49	89,1%
1	6	10,9%
**Receptor status**	**N**	
ER+/PR+/Her2–	30	54,5%
Triple positive	4	7,3%
Triple negative	11	20,0%
ER+/PR-/Her2–	7	12,7%
ER–/PR+/Her2–	1	1,8%
ER–/PR–/Her2+	2	3,6%

Table shows numbers and age of patients with breast cancer, benign breast diseases and healthy controls as well as different clinical features of breast cancer patients (UICC-, T-, N-, M-stage and receptor status).

Written informed consent was obtained from all patients for blood collection in the Biofluid Biobank of the University Hospital Bonn at the Institute for Clinical Chemistry and Clinical Pharmacology supported by the Center for Integrated Oncology Cologne-Bonn (CIO). This process as well as the use of the samples for the planned study were approved by the Local Ethics Committee of the University Bonn.

Blood samples were collected prospectively between 2010 and 2013 during a residence or ambulant consultations at the Department for Gynecology and Obstetrics of the University Hospital Bonn simultaneously with the routine blood samplings. They were immediately transported to the Central Laboratory of the University Hospital Bonn and centrifuged at 4000 rpm (3300 G) for 10 minutes. Subsequently, serum samples were aliquoted into polypropylene vials and archived at −80°C in the biobank store, labeled with a double-pseudonomized code. Corresponding to the code the pathology report and clinical history of each patient was documented in detail.

### Methods

RNA-Isolation was done from 400 μL serum using the mirVana PARIS Kit (Ambion, Foster City, CA, USA) after the addition of 400 μl 2× denaturation buffer. After short vortexing and a short storage on ice, 5 μl of the synthetic cel-miR-39 miScript miRNA Mimic (Qiagen, Hilden, Germany) in the concentration of 5 fmol/μl was added. The organic extraction started by adding 800 μl acid-phenol/chloroform to each sample. The samples were short mixed and centrifuged at 4000 rpm for 15 minutes by room-temperature. Then a minimum of 500 μl of the upper aqueous phase was taken into another tube. These tubes were centrifuged at 4000 rpm for 5 minutes and 500 μl were decanted in another tube. To each sample 1.25 volumes of ethanol (100%) were added followed by 3 steps of filtration with two different wash solutions. Finally, miRNA was eluted in 50 μl elution solution. The whole procedure was performed according to the manufacturer´s protocol. Subsequently, every sample was measured by spectro-photometry in a Tecan Infinite M200 Pro NanoQuant (Crailsheim, Germany).

Reverse transcription was carried out with the miScript Reverse Transcription Kit (Qiagen, Hilden, Germany). 12 μl isolated RNA, 4 μl miScript RT Buffer (5× HiFlex), 2 μl 10× miScript Nucleics Mix and 2 μl miScript Reverse Transcriptase Mix were used for the reverse transcription. The solution was incubated in the LifeTouch Thermal Cycler (Biozym, Scientific GmbH, Oldendorf, Germany) at 37°C for 60 minutes and at 95°C for 5 minutes. Final elution volume was 20 μL, which was diluted with 80 μl RNAse-free water. The aliquots were stored at –20°C for further analysis or maintained on ice for immediate rtPCR.

Quantitative real-time-PCR was carried out using the miScript SYBR Green PCR Kit (Qiagen, Hilden, Germany) with predesigned miScript PCR primers (Qiagen). Details to the single miRNA primer sequences and assays are given in the Supplementary Table 1 (Supplementary Table 1). RtPCR-quantification of micro-RNA was performed using target-specific miScript Primer Assays (forward primers) and the miScript SYBR Green PCR Kit that contains the miScript Universal Primer (reverse primer) and QuantiTect SYBR Green PCR Master Mix (Qiagen) according to the manufacturer's protocol. 10 μl 2× QuantiTect SYBR Green PCR MasterMix, 2 μl 10× Universal-Reverse-Primer, 2 μl miScript-PCR Primer Assay, 4 μl RNAse-free water and 2 μL reverse transcription product were used for the rtPCR with each sample and were pipetted into 96-well plates (Roche Applied Sciences, Basel, Switzerland). All rtPCR experiments were carried out on a LightCycler 480 (Roche) using the following conditions: 95°C for 15 minutes followed by quantitative real-time PCR amplification, which was conducted by 40 cycles of denaturation (94°C for 15 s), annealing (55°C for 30 s) and extension (70°C for 30 s).

Details of method establishment, validation and quality controls were charted in accordance with the MIQE guidelines. Standards for all assays were produced by a SKBR cell line that was diluted until 1:10E6. Melting curve analysis confirmed specificity of the PCR products. All samples calibrators and controls were measured as double determinations and patient groups were mixed in all plates to minimize the inter-assay variability. 5 fmol/μl of the synthetic cel-miR-39 miScript miRNA Mimic (Qiagen, Hilden, Germany) was spiked in all samples before RNA-isolation to control extraction and reverse transcription efficiency and quantification. MiR-16 was used as an endogenous control to normalize the micro-RNA expression. Potential blood contamination by hemolysis was controlled by the ratio of delta-Ct of miR-451 and miR-23a [32]. The relative quantity of each miRNA was determined using the comparative Ct method (LightCycler 480 RT-PCR Instruments, Roche) and miR-16 as reference miRNA [28, 34].

Tumor markers were measured by automated chemiluminescence immunoassays on Dimension Vista 1500 systems (Siemens Healthineers, Erlangen, Germany) for CEA, CA 15-3 and CA 19-9 and on the Architect i1000 SR platform (Abbott Diagnostics, Wiesbaden, Germany) for CA 125.

### Statistics

Performances of RNA quantity, single miRNAs and tumor markers were assessed for the discrimination between breast cancer patients and healthy controls as well as between breast cancer patients and patients with benign breast diseases. In addition, subgroup analyses for association of the biomarkers with UICC-stage, T-stage, N-stage, ER- and PR-receptor status were done. After normalization by celmir-39 and miR-16, miRNAs, RNA quantity and also tumor marker levels were log-transformed for variance stabilization. Significance testing was done using *t*-Test or Wilcoxon rank sum test when data were not following a normal distribution. As the evaluation had explorative character, no adjustment for multiple testing was performed. Differences with a *p*-level of *p* ≤ 0.05 were considered significant. In addition, areas under the curve (AUC) of receiver operating characteristic (ROC) curves were calculated and sensitivities of relevant biomarkers at 95% specificity versus the control groups were given.

## CONCLUSIONS

Our findings show a promising performance of miR-34a for the differential diagnosis and staging of breast cancer. Most importantly, the combination with established tumor markers CEA or CA 15-3 improved the sensitivity for breast cancer detection suggesting further validation studies with this combined biomarker approach.

## SUPPLEMENTARY MATERIALS FIGURES AND TABLES


