# Supplementary material for: Improved sensitivity for detection of breast cancer by combination of miR-34a and tumor markers CA 15-3 or CEA
Source: Oncotarget. 2018 Apr 27;9(32):22523–36. doi: 10.18632/oncotarget.25077 (PMC5976482; doi:10.18632/oncotarget.25077)
Supplement: Supplementary file 1 [file oncotarget-09-22523-s001.pdf]

Improved sensitivity for detection of breast cancer by combination of miR-34a and tumor markers CA 15-3 or CEA

SUPPLEMENTARY MATERIALS

Supplementary Table 1: Details and catalogue numbers of kits and miRNA- primer assays

Item	Target	Sequence	Catalogue number	Company
mirVana Isolation Kit Paris			AM1556	Ambion
miScript II Reverse Transcription Kit			218161	Qiagen
Qiagen miScript Sybr Green			218073	Qiagen
Hs_miR-16_1 miScript Primer Assay	hsa-miR-16-5p	MIMAT0000069: 5′UAGCAGCACGUAAAUAUUGGCG	MS00006517	Qiagen
Hs_miR-21_2 miScript Primer Assay	hsa-miR-21-5p	MIMAT0000076: 5′UAGCUUAUCAGACUGAUGUUGA	MS00009079	Qiagen
Hs_miR-23a_2 miScript Primer Assay	hsa-miR-23a-3p	MIMAT0000078: 5′AUCACAUUGCCAGGGAUUUCC	MS00031633	Qiagen
Hs_miR-34a_1 miScript Primer Assay	hsa-miR-34a-3p	MIMAT0004557: 5′CAAUCAGCAAGUAUACUGCCCU	MS00009534	Qiagen
Hs_miR-92a_1 miScript Primer Assay	hsa-miR-92a-3p	MIMAT0000092: 5′UAUUGCACUUGUCCCGGCCUGU	MS00006594	Qiagen
Hs_miR-155_2 miScript Primer Assay	hsa-miR-155-5p	MIMAT0000646: 5′UUAAUGCUAAUCGUGAUAGGGGU	MS00031486	Qiagen
Hs_miR-222_2 miScript Primer Assay	hsa-miR-222-3p	MIMAT0000279: 5′AGCUACAUCUGGCUACUGGGU	MS00007609	Qiagen
Hs_miR-451_1 miScript Primer Assay	hsa-miR-451a	MIMAT0001631: 5′AAACCGUUACCAUUACUGAGUU	MS00004242	Qiagen
Hs_let-7c_1 miScript Primer Assay	hsa-let-7c-5p	MIMAT0000064: 5′UGAGGUAGUAGGUUGUAUGGUU	MS00003129	Qiagen
Syn-cel-miR-39-3p miScript miRNA Mimic	cel-miR-39-3p	MIMAT0000010: 5′UCACCGGGUGUAAAUCAGCUUG	MSY0000010	Qiagen
